# A nationwide questionnaire survey to investigate facility-based disparities in satisfaction and working conditions of surgical trainees in Japan: university hospitals, community hospitals, and hybrid-type facilities

**DOI:** 10.1007/s00595-025-03081-3

**Published:** 2025-06-26

**Authors:** Daisuke Koike, Kosei Takagi, Keisuke Arai, Yoshiyuki Kiyasu, Takashi Kohmura, Chiaki Suda, Shinkichi Takamori, Wataru Takayama, Mai Nakamura, Masayuki Fukumoto, Yoshiko Yamaoka‐Fujikawa, Genki Watanabe, Jun Watanabe, Saseem Poudel, Norihiko Ikeda, Akinobu Taketomi, Mitsue Saito

**Affiliations:** 1 Department of Gastroenterological Surgery, Fujita Health University Bantane Hospital, Nagoya, Japan; 2 Japan Surgical Society Education Committee, Japan Surgical Society, Tokyo, Japan; 3 Department of Gastroenterological Surgery, Okayama University Graduate School of Medicine, Dentistry, and Pharmaceutical Sciences, 2-5-1 Shikata-Cho, Kita-Ku, Okayama, 700-8558 Japan; 4 Department of Surgery, Division of Hepato-Biliary-Pancreatic Surgery, Kobe University Graduate School of Medicine, Hyogo, Japan; 5 Department of Surgery, Kyoto University Graduate School of Medicine, Kyoto, Japan; 6 Department of Surgery, Fujisawa Shounandai Hospital, Kanagawa, Japan; 7 Department of Public Health, Gunma University Graduate School of Medicine, Gunma, Japan; 8 Department of Surgery and Science, Graduate School of Medical Sciences, Kyushu University, Fukuoka, Japan; 9 Department of Thoracic and Breast Surgery, Faculty of Medicine, Oita University, Oita, Japan; 10 Department of Emergency and Disaster Medicine, Tokyo Medical and Dental University, Tokyo, Japan; 11 Department of Surgery, National Center for Global Health and Medicine, Tokyo, Japan; 12 Department of Surgery, Nagasaki University Graduate School of Biomedical Sciences, Nagasaki, Japan; 13 Department of Upper Gastrointestinal Surgery, Kitasato University School of Medicine, Kanagawa, Japan; 14 Department of Hepato-Biliary-Pancreatic Surgery, Osaka Metropolitan University Graduate School of Medicine, Osaka, Japan; 15 Department of Surgery, Division of Gastroenterological, General and Transplant Surgery, Jichi Medical University, Tochigi, Japan; 16 Department of Gastroenterological Surgery II, Hokkaido University, Sapporo, Japan; 17 Japan Surgical Society, Tokyo, Japan; 18 Department of Surgery, Tokyo Medical University, Tokyo, Japan; 19 Department of Gastroenterological Surgery I, Hokkaido University Graduate School of Medicine, Hokkaido, Japan; 20 Department of Breast Oncology, Juntendo University Graduate School of Medicine, Tokyo, Japan

**Keywords:** Surgical training, Disparity, Work–life integration, Training satisfaction

## Abstract

**Purpose:**

To investigate disparities and problems within surgical training programs in Japan based on the type of host facility.

**Methods:**

A post hoc analysis of a nationwide questionnaire survey was performed to evaluate program outcomes and working conditions among university hospitals (Group U), a combination of university and community hospitals (Group UC), and community hospitals (Group C).

**Results:**

A total of 758 respondents were analyzed across Groups U (*n* = 199), UC (*n* = 299), and C (*n* = 260). Groups U and UC had lower satisfaction scores and smaller surgical volumes than Group C. Academic activity and nonsurgical training in Group U were not superior to those in the other groups. Although overtime work did not differ significantly among groups, poorer working conditions, including a lower rate of overtime allowance, more frequent night duties, and a lower annual income, were found in Group U than in the other groups.

**Conclusion:**

This study revealed disparities and problems in training programs based on the type of host facility. Further improvements in working conditions and educational contexts are expected to help increase satisfaction levels and recruitment of surgical residents in the future.

## Introduction

In recent decades, the number of surgeons in Japan has decreased, despite the fact that the total number of physicians in Japan has increased approximately 1.3-fold since 2000 [1]. The number of applicants for surgical training programs has also decreased in recent years, although the Japanese Surgical Society (JSS) has recently revised the surgical training system for board-certified surgeons [2]. To clarify the status and issues concerning the current surgical training system in Japan, the JSS Educational Committee conducted a comprehensive nationwide online survey [3], which revealed a high satisfaction rate with the overall surgical training program but also identified several specific problems related to the trainees’ working conditions and harassment.

Surgical trainees in Japan are required to complete a 3 year training program and rotation at the main and secondary institutions for a minimum of 6 months each [4]. Surgical training programs in Japan can be divided into three types based on the type of host facility: university hospital, community hospital, or a combination of both (also called hybrid type). University hospitals tend to perform highly specialized procedures, handle complicated cases, and engage in academic activities. Community-based hospitals tend to perform more common procedures with high commitment from trainees and substantial support during surgeries. Trainees were asked to choose a training facility based on their career preferences.

A previous nationwide study conducted among Japanese surgery trainees who graduated in 2016 revealed disparities and problems between university and community-based training programs in terms of training duration and surgical volume [5]. These findings led to a revision in training programs by the JSS intended to improve facility-based training program disparities; however, no survey has yet investigated the outcome of the implemented changes.

In this post hoc analysis of a nationwide questionnaire survey conducted in 2023 [3], we investigated the current host facility-based disparities within surgical training programs in Japan. The findings will help enable facilities and instructors to further improve the training programs and match them with trainee expectations.

## Methods

### Study settings

Regarding surgical training systems for board-certified surgeons in Japan, after a 2 year intern program, trainees apply for a surgical training program provided by each institution or surgical department. The current training program for board-certified surgeons requires a minimum of 3 years of training, which includes at least 6 months of training each at a primary and a secondary institution (the trainee should be trained at a minimum of two institutions). The minimum required surgical volumes were 120 procedures performed by primary surgeons and 350 procedures performed by either the primary or assisting surgeon.

### Survey

The Under 40 working group of the JSS Educational Committee developed a questionnaire survey to clarify the current status of the training program and the challenges faced by trainees. Details of the survey methods have been reported previously [3]. The survey questions pertained to demographic information, institutional information, training duration at each type of institution (university or community hospital), and other variables including program outcomes and working conditions. A four-point Likert scale was used with a relative questionnaire. Due to the COVID-19 pandemic, certified examinations have been extended to 2021. Therefore, the survey was conducted with 2021 and 2022 graduates of the surgical training program.

### Data collection and statistical analyses

Participants in a typical training program were divided into three groups: trained mainly at a university hospital (Group U), trained at a combination of both university and community hospitals (Group UC), and trained mainly at a community hospital (Group C). Each group was defined as follows: Group U included trainees with the longest duration of training at a university hospital; Group C included trainees with the longest duration of training at a community hospital and with no more than 6 months of university-based training; and Group UC included those with the longest duration of training at a community hospital but trained for more than 7 months at a university hospital. The types of programs were defined according to the trainees’ declarations in the questionnaire. Differences between the three groups were analyzed using the Mann–Whitney *U* test, Kruskal–Wallis test, or Chi-square test. The post hoc test was performed using the Bonferroni correction. Statistical significance was set at *p* < 0.05. All statistical analyses were performed using the IBM SPSS Statistics software program, version 28 (IBM Corporation, Armonk, NY, USA).

### Ethical approval

This study was approved by the Research Ethics Review Committee of the Japanese Surgical Society (JSS2023-1). Consent for participation in the survey was obtained from all participants before the questionnaire was administered. No personal data were obtained, to protect the respondents’ anonymity.

## Results

### Demographic background of the responding surgical trainees

After excluding 125 respondents with incomplete data sets, the available survey response rate was 53.8% (758/1410). In total, 758 respondents were included in this study. Group U included 199 trainees, Group UC included 299 trainees, and Group C included 260 trainees. The demographic characteristics of each group are presented in Table 1. Post-graduate years, age, sex, marital status, and having a child did not differ markedly among the three groups. Groups U and C had fewer trainees who were trained in a regional or rural area than did Group UC. While most of the trainees were interested in gastroenterological surgery, Group U had a higher proportion of trainees with an interest in other subspecialties.

**Table 1:** Demographic background of the responding surgical trainees by host facility

	Group U (*n* = 199)	Group UC (*n* = 299)	Group C (*n* = 260)	*p* Value^a^
Post-graduate (years)				
6	95 (47.7)	114 (38.1)	106 (40.8)	0.14
7	72 (36.2)	142 (47.5)	121 (46.5)	
Others	31 (15.6)	42 (14.0)	30 (11.5)	
Age (years)				
≤ 30	34 (17.1)	40 (13.4)	43 (16.5)	0.22
31–35	144 (72.4)	228 (76.3)	201 (77.3)	
36–40	19 (9.5)	27 (9.0)	11 (4.2)	
> 40	2 (1.0)	4 (1.3)	5 (1.9)	
Sex				
Male	135 (67.8)	230 (76.9)	199 (76.5)	0.09
Female	64 (32.2)	69 (23.1)	61 (23.5)	
Marital status				
Married/with partner	140 (70.4)	212 (70.9)	189 (72.7)	0.37
Have child(ren)	80 (40.2)	121 (40.5)	112 (43.1)	0.65
Training area				
Urban	91 (45.7)	107 (35.8)	120 (46.2)	0.007
Regional city	104 (52.3)	173 (57.9)	133 (51.2)	
Rural	4 (2.0)	19 (6.4)	6 (2.3)	
Subspecialty of interest				
Gastroenterological surgery	84 (42.2)	167 (55.9)	148 (56.9)	0.02
Cardiovascular surgery	32 (16.1)	33 (11.0)	21 (8.1)	
Thoracic surgery	25 (12.6)	32 (10.7)	28 (10.8)	
Breast surgery	31 (15.6)	30 (10.0)	24 (9.2)	
Others	27 (13.6)	36 (12.0)	38 (14.6)	

Group U trained mainly at a university hospital, Group UC hybrid type: trained at a combination of university/community hospital, Group C trained mainly at a community hospital. Data are number (percentage)

^a^Chi-square test

### Program outcomes

**Fig. 1:**
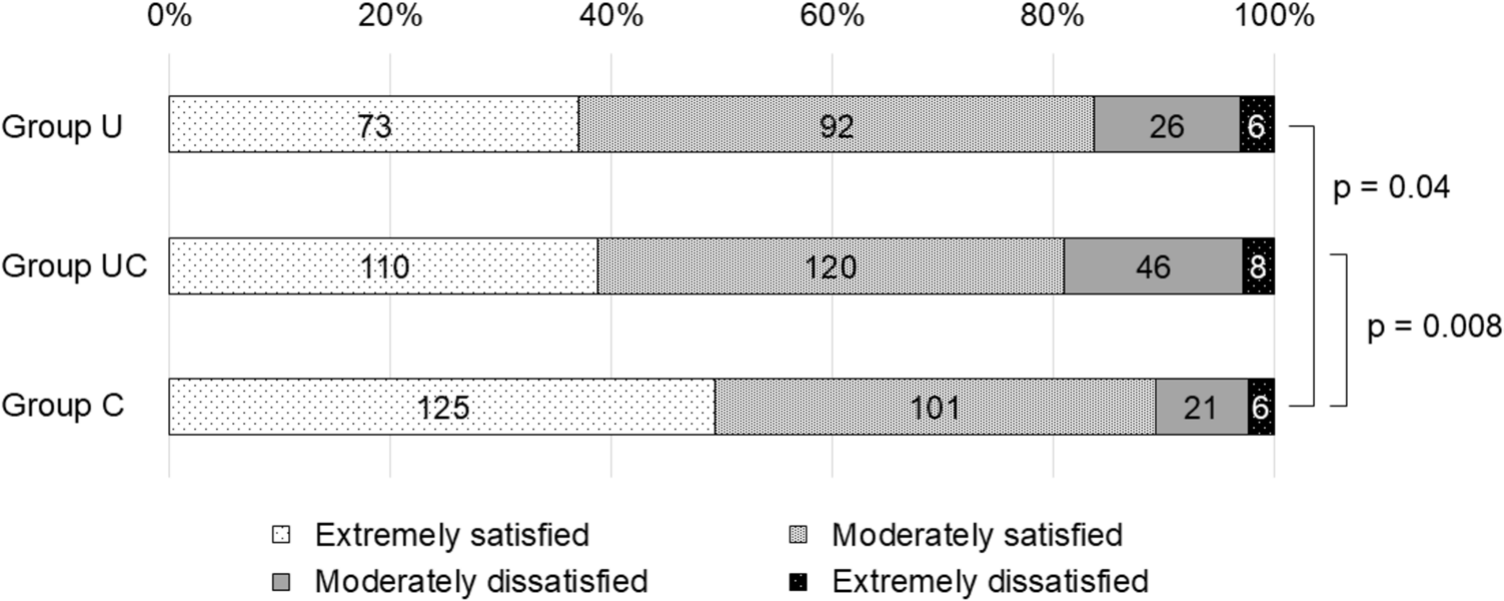
Satisfaction with the surgical training program. The number in the fields indicates the number of respondents. A Kruskal–Wallis test with Bonferroni correction was used to compare the three groups. Group U, trained mainly at a university hospital; Group UC [hybrid type], trained at a combination of university/community hospital; Group C, trained mainly at a community hospital

**Fig. 2:**
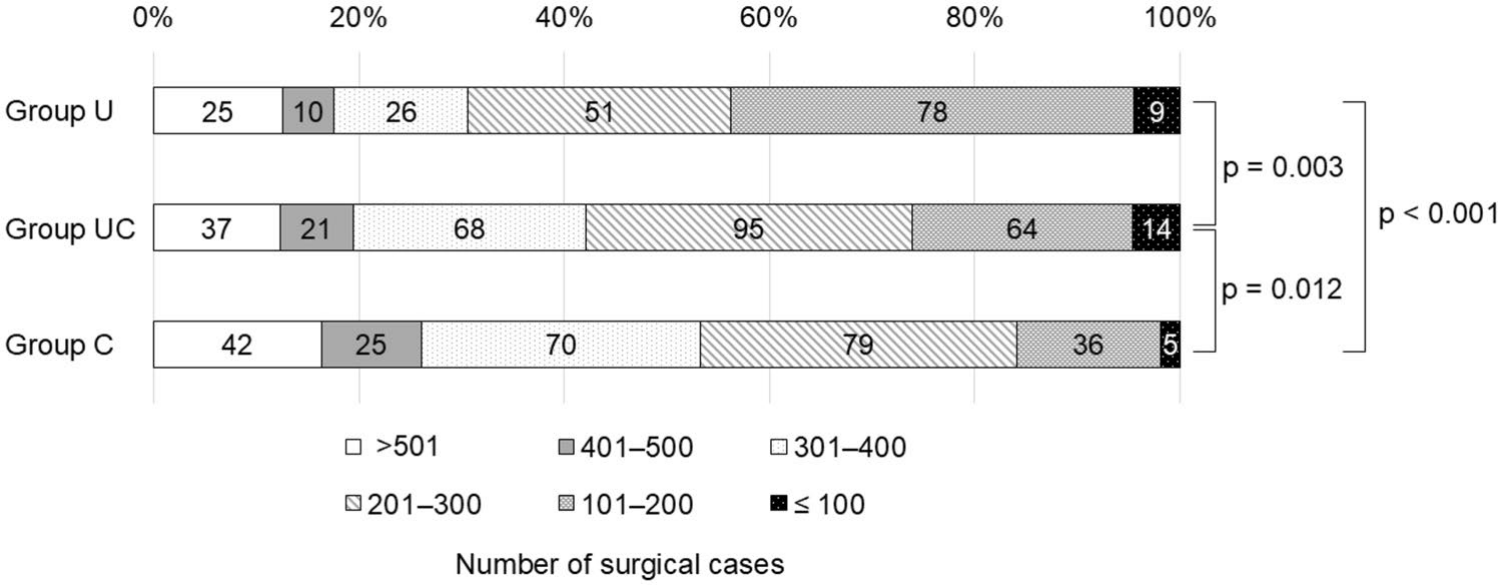
Surgical volume during the training period. The numbers in the fields indicate the numbers of respondents. A Kruskal–Wallis test with Bonferroni correction was used to compare the three groups. Group U, trained mainly at a university hospital; Group UC [hybrid type], trained at a combination of university/community hospital; Group C, trained mainly at a community hospital

The total satisfaction with the training program was over 80%. However, Groups U and UC reported lower satisfaction rates than Group C (*p* = 0.04, p = 0.008, respectively) (Fig. 1). Groups U and UC had less surgical experience than Group C (*p* < 0.001 and *p* = 0.01, respectively) (Fig. 2). Approximately 44% of Group U experienced fewer than 200 cases during the training program, while 26% of Group UC and 16% of Group C experienced fewer than 200 cases.

The evaluations of instructors, including clinical skills (*p* = 0.63) and teaching skills (*p* = 0.58), were not significantly different between the groups (Table 2). However, instructors’ teaching skills were rated more poorly than their clinical skills in all the groups (*p* < 0.001). Participation in off-the-job training for technical skills at least once during the training program was approximately 85% in each group; however, participation in off-the-job training for non-technical skills was only 30%, and these proportions did not differ markedly among groups. The number of papers published during training in Group U was not higher than that in the other groups (*p* = 0.59). The limitations of submitting papers were similar across all three groups. For trainees, the main reasons why they find it difficult to submit papers are that they have not been taught the know-how on writing and submission in all three groups, followed by a lack of time for writing in Groups U and C, and a lack of motivation or interest in writing in Group UC (Table 2). Respondents in Groups U and UC reported a lack of clinical knowledge (p = 0.048), and respondents in Groups U and C reported a lack of mentorship (*p* = 0.03) as a limitation for writing.

**Table 2:** Program outcomes by host facility

	Group U (*n* = 199)	Group UC (*n* = 299)	Group C (*n* = 260)	*p* Value^a^
Satisfaction with instructors’ clinical skills				
Extremely satisfied	83 (41.7)	128 (42.8)	120 (46.2)	0.63
Moderately satisfied	107 (53.8)	148 (49.5)	125 (48.1)	
Moderately dissatisfied	7 (3.5)	4 (1.3)	9 (3.5)	
Extremely dissatisfied	0 (0)	4 (1.3)	0 (0)	
Satisfaction with instructors’ teaching skills				
Extremely satisfied	61 (30.7)	86 (28.8)	80 (30.8)	0.58
Moderately satisfied	106 (53.3)	155 (51.8)	130 (50.0)	
Moderately dissatisfied	27 (13.6)	36 (12.0)	34 (13.1)	
Extremely dissatisfied	3 (1.5)	7 (2.3)	10 (3.8)	
Number of published papers				
0	44 (22.1)	65 (21.7)	60 (23.1)	0.59
1	74 (37.2)	84 (28.1)	83 (31.9)	
2	38 (19.1)	57 (19.1)	41 (15.8)	
3	18 (9.0)	33 (11.0)	31 (11.9)	
4	8 (4.0)	15 (5.0)	19 (7.3)	
≥ 5	15 (7.5)	29 (9.7)	19 (7.3)	
Limitations to publishing a paper (multiple choice question)				
Methods to write or submit papers	129 (64.8)	172 (57.5)	152 (58.5)	0.23
Time for writing	100 (50.3)	123 (41.1)	123 (47.3)	0.11
Clinical knowledge	80 (40.2)	113 (37.8)	78 (30.0)	0.048
Motivation or interest	75 (37.7)	134 (44.8)	108 (41.5)	0.28
Data, cases	71 (35.7)	92 (30.8)	78 (30.0)	0.39
Mentor	64 (32.2)	71 (23.7)	86 (33.1)	0.03
Colleague for writing papers	32 (16.1)	54 (18.1)	45 (17.3)	0.85
Nothing	1 (0.5)	2 (0.7)	4 (1.5)	0.43
Off-the job training, technical skills				
Regularly	36 (18.1)	38 (12.7)	53 (20.4)	0.17
Irregularly	135 (67.8)	212 (70.9)	176 (67.7)	
Not at all	26 (13.1)	34 (11.4)	25 (9.6)	
Off-the job training, non-technical skills				
Regularly	12 (6.0)	18 (6.0)	20 (7.7)	0.94
Irregularly	47 (23.6)	65 (21.7)	57 (21.9)	
Not at all	138 (69.3)	199 (66.6)	177 (68.1)	
Problems with the training program (multiple choice question)				
Nothing	73 (36.7)	113 (37.8)	104 (40.0)	0.75
Mismatch of training content and target	52 (26.1)	74 (24.7)	65 (25.0)	0.94
Curriculum content not understood by trainees	58 (29.1)	63 (21.1)	54 (20.8)	0.06
Inadequate feedback from instructors	29 (14.6)	42 (14.0)	53 (20.4)	0.10
Training program of the facility is not evaluated	19 (9.5)	47 (15.7)	29 (11.2)	0.09
Attainment of achievement goals is not evaluated	26 (13.1)	22 (7.4)	21 (8.1)	0.07

Group U trained mainly at a university hospital, Group UC hybrid type: trained at a combination of university/community hospital, Group C trained mainly at a community hospital. Data are number (percentage)

^a^Chi-square test

Regarding problems with the training program, approximately 60% of respondents in each group pointed out problems. The most common problem was ‘curriculum content not understood by trainees’ in Group U and ‘mismatch of training content and target’ in Groups UC and C (Table 2).

### Working conditions

**Fig. 3:**
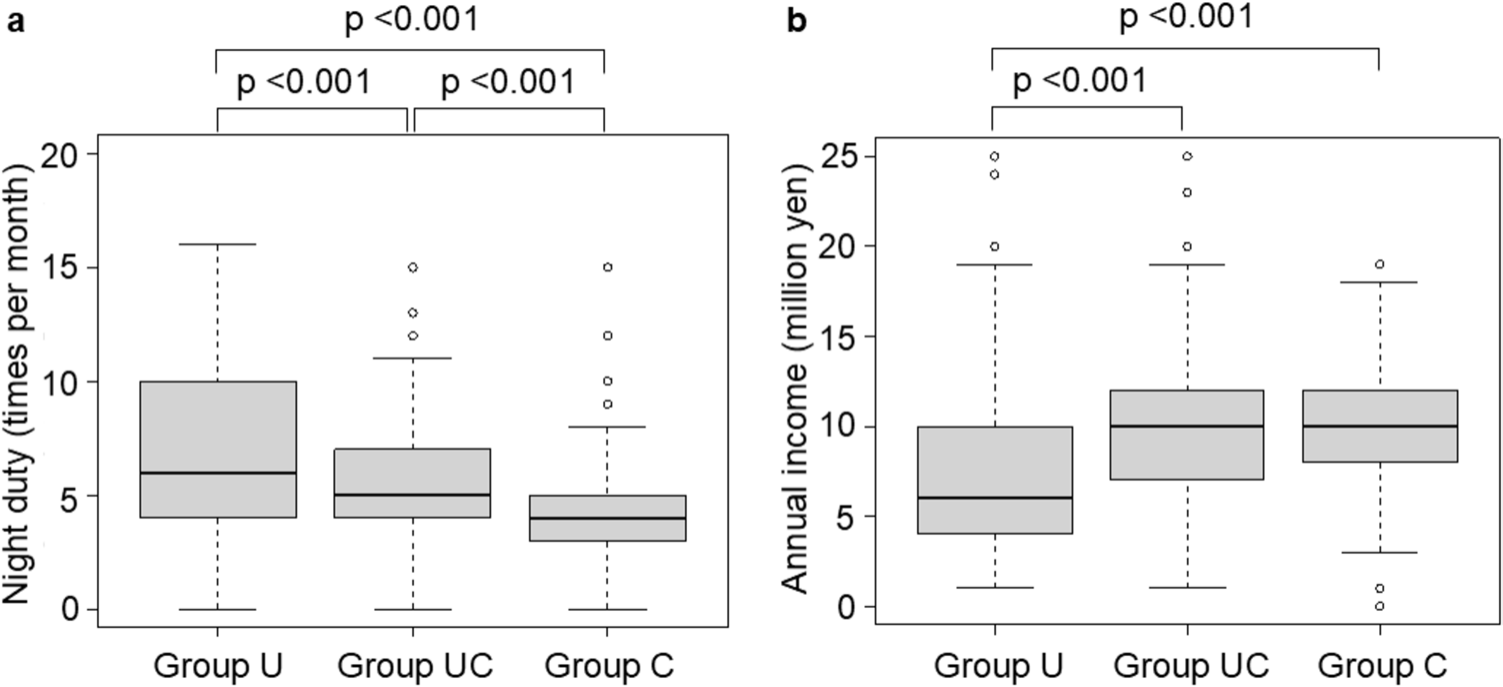
Box plots of (**a**) night duty during the training program and (**b**) annual income during the last training year. In **a**, each data point represents number of times night duty was performed per month. Lower and upper fences are 25 and 75th percentiles, and the bold horizontal line is the median. Bars represent the 10 and 90th percentiles. Dots mark outliers. A Kruskal–Wallis test with Bonferroni correction was used to compare the three groups. In **b**, each data point represents the last annual income from training facilities. Lower and upper fences are 25 and 75th percentiles, and the bold horizontal line is the median. Bars represent the 10 and 90th percentiles. Dots mark outliers. A Kruskal–Wallis test with Bonferroni correction was used to compare the three groups. Group U, trained mainly at a university hospital; Group UC [hybrid type], trained at a combination of university/community hospital; Group C, trained mainly at a community hospital

Working hours in Group U (20.6%) tended to be strictly recorded compared to those in Groups UC (8.7%) and Group C (13.5%) (*p* < 0.001; Table 3). The median number of night shifts per month was 6 (interquartile range [IQR], 4–10) in Group U, which was higher than the 5 (IQR, 4–7) in Group UC and 4 (IQR, 3–5) in Group C (*p* < 0.001, *p* < 0.001, respectively, Fig. 3a).

**Table 3:** Working conditions by host facility

	Group U (*n* = 199)	Group UC (*n* = 299)	Group C (*n* = 260)	*p* Value^a^
Working hour management				
Strictly recorded	41 (20.6)	26 (8.7)	35 (13.5)	< 0.001
Roughly recorded	86 (43.2)	178 (59.5)	147 (56.5)	
Not recorded	64 (32.2)	73 (24.4)	66 (25.4)	
Overtime work per month				
≤ 80 h	65 (32.7)	89 (29.8)	78 (30.0)	0.34
81–160 h	94 (47.2)	159 (53.2)	132 (50.8)	
> 160 h	21 (10.6)	23 (7.7)	33 (12.7)	
Correspond to overtime allowance (multiple choice question)				
Surgery or clinical care	141 (70.9)	239 (79.9)	234 (90.0)	< 0.001
Surgery-related work^b^	50 (25.1)	99 (33.1)	121 (46.5)	< 0.001
Conference participation	43 (21.6)	65 (21.7)	101 (38.8)	< 0.001
Preparing for outpatients or conferences, making surgical records	26 (13.1)	58 (19.4)	80 (30.8)	< 0.001
Academic activities	4 (2.0)	10 (3.3)	15 (5.8)	0.10
No compensation for overtime work	36 (18.1)	15 (5.0)	7 (2.7)	< 0.001
Experience of harassment by instructors				
Yes	85 (42.7)	115 (38.5)	104 (40.0)	0.78

Group U trained mainly at a university hospital, Group UC hybrid type: trained at a combination of university/community hospital, Group C trained mainly at a community hospital. Data are number (percentage)

^a^Chi-square test

^b^Surgery-related work includes specimen sorting and similar tasks

Overtime work, in addition to regular working hours, was similar among the three groups (*p* = 0.34). However, 57.8% of Group U, 60.9% of Group UC, and 63.5% of Group C experienced over 80 h of overtime work per month. Group U had the lowest correspondence to overtime allowance for all tasks and the highest rates of no compensation for overtime work (Table 3).

The percentage of respondents who subjectively reported that they had experienced behaviors that could potentially qualify as harassment by instructors was not significantly different among groups (U 42.7%, UC 38.5%, C 40.0%, *p* = 0.78) (Table 3).

The annual income for each group during the last training year is shown in Fig. 3b. The median annual income of Group U was 6 million yen (equal to approximately 38,710 USD, based on an exchange rate of 1 USD = 155 JPY) (IQR, 4–10), which is significantly lower than the 10 million yen (about 64,516 USD) (IQR, 7–12; *p* < 0.001) in Group UC and 10 million yen (IQR, 8–12; *p* < 0.001) in Group C. The incomes of Groups UC and C were not markedly different.

## Discussion

The present post hoc analysis of a nationwide survey of surgical training program graduates in 2021 and 2022 demonstrated the impact of the type of host facility on the satisfaction and working conditions of surgical residents in Japan. Although a similar demographic background was found for graduates of the training programs in all groups, Group U reported a lower satisfaction, lower rate of surgical exposure, and poorer compensation status.

Regarding program outcomes, although satisfaction scores were relatively high in all three groups, Group U and UC had lower satisfaction scores than Group C. Several factors influencing surgical training programs have been reported, such as surgical volume and variety, educational context including intraoperative and didactic out-of-theater training, adequate relationship with mentors, social status of facilities, and trainee characteristics [6–10]. This study found no significant differences in the evaluation of instructors, research activities, or off-the-job training among the groups; hence, these variables cannot explain the lower satisfaction in Groups U and UC than in Group C. In contrast, a significantly lower rate of surgical exposure was found in Group U than in Groups UC and C. More surgical experience could potentially improve satisfaction scores and enhance competencies in Group U. Since the volume of surgical exposure reflects the surgical competency of the resident, an appropriate number of surgical cases is recommended to enhance surgical competency [11].

Regarding working conditions, we found that approximately 60% of surgical residents in each group worked more than 80 h of overtime work per month. Furthermore, working hour management and overtime allowance differed significantly among the groups. Notably, Group U had the highest number of night duty shifts per month and the lowest annual income. Poor working conditions, combined with insufficient compensation, are possible additional factors that could explain the lower satisfaction in Group U. The working conditions of residents, particularly those in Group U, should be improved. Task shifting and institutional support for surgical residents are urgently needed to increase the number of residents. The recent work style reform for physicians in Japan, which limits overtime, presents significant challenges to surgical training. These are challenges that many countries have already faced and addressed from the perspective of trainee well-being [12, 13]. Given that traditional surgical training relies heavily on long working hours to accumulate experience, it is necessary to prioritize more efficient use of time, implement well-structured off-the-job training, and expand simulation-based education to maintain the training quality while complying with the new work style reform regulations.

The various roles of hospitals differ, as university hospitals have a mission to provide advanced medical care, education, and research. Several advantages of university hospitals over others, including human resources and educational contexts, are effective in improving training programs and increasing trainee satisfaction. However, surgical experience at university hospitals may be limited for trainees because of their role in providing advanced medical care. The present study confirmed lower surgical volumes for trainees at university hospitals, as previously reported for Japan [5, 14]. By contrast, trainees at community-based facilities have the opportunity to treat relatively common diseases. It is recommended that university hospitals enhance the surgical training program first and foremost by increasing the surgical experience of trainees. Second, the low surgical exposure rate can be improved, such as by offering more didactic lectures and off-the-job training and by increasing the educational competency of instructors. The centralization of medical institutions has been considered a potential countermeasure to enhance surgical exposure for trainees, given the declining population and large number of surgical departments in Japan. Centralization would not only concentrate surgical experience for trainees but also improve surgical outcome for patients [15, 16]. However, implementing such centralization may present challenges, including accessibility to surgical care and the need to restructure healthcare delivery systems. The centralization of educational lectures, the introduction of remote simulation training such as virtual scenarios, and the development of remote mentoring systems are also being explored as potential strategies to address the challenges posed by a declining population, as well as the need to reduce costs and efforts [17, 18]. Future studies should examine strategies to balance the enhancement of surgical training opportunities while ensuring equitable access to care.

The problems of the training program were similar across the three groups, suggesting that these problems originated from national-level organizations rather than institution-level factors. Assessment systems for surgical curricula have already been implemented in some countries to achieve appropriate surgical education, including observational and self-assessment with feedback [19–22]. The current surgical training program in Japan lacks an assessment system for surgical competency in the interim or completion stages. Since trainees are trained at a minimum of two facilities under the new program, without any systematic assessment methods, program directors would inevitably have difficulties maintaining continuity for the total number of surgical exposures and adjusting the variety of surgeries for each trainee. A"train-the-trainer"system is also necessary for the systematic assessment of trainees; however, it is currently lacking in the surgical training system in Japan. This deficiency may be reflected in the observed gap between the educational and clinical skills of the instructors in this study. Nevertheless, initiatives to develop training for trainers in the surgical field are now emerging, as promoted by the recently established Japanese Association for Surgical Education [23]. Additionally, resources, such as time, incentives, and access to appropriate teaching materials and tools, are essential for the successful implementation of a training-the-trainer system and, ultimately, to ensure the quality of trainee education [24]. The implementation of strategic systems for the assessment of the quantitative and qualitative aspects of a training program is also vital for trainees to achieve individual goals.

Several limitations associated with the present study warrant mention. First, trainees chose the training facility based mainly on their preferences and expectations, and the results may have been affected by selection bias. Second, this questionnaire survey was conducted only with trainees and did not include all aspects of the program.

In conclusion, this study revealed disparities among training programs at university-based, community-based, and hybrid-type host facilities. Although we found relatively high satisfaction levels in all groups, differences in surgical volume, overtime allowance status, and annual income were found between university and community-based programs. Improving current shortcomings and strengthening existing merits at each host facility are expected to narrow disparities, increase satisfaction levels, and increase the number of applications for future surgical training programs.
