# Correction: A nationwide questionnaire survey to investigate facility‑based disparities in satisfaction and working conditions of surgical trainees in Japan: university hospitals, community hospitals, and hybrid‑type facilities

**DOI:** 10.1007/s00595-025-03160-5

**Published:** 2025-11-14

**Authors:** Daisuke Koike, Kosei Takagi, Keisuke Arai, Yoshiyuki Kiyasu, Takashi Kohmura, Chiaki Suda, Shinkichi Takamori, Wataru Takayama, Mai Nakamura, Masayuki Fukumoto, Yoshiko Yamaoka‐Fujikawa, Genki Watanabe, Jun Watanabe, Saseem Poudel, Norihiko Ikeda, Akinobu Taketomi, Mitsue Saito

**Affiliations:** 1 Department of Gastroenterological Surgery, Fujita Health University Bantane Hospital, Nagoya, Japan; 2 Department of Gastroenterological Surgery, Okayama University Graduate School of Medicine, Dentistry, and Pharmaceutical Sciences, 2-5-1 Shikata-Cho, Kita-Ku, Okayama, 700-8558 Japan; 3 Department of Surgery, Division of Hepato-Biliary-Pancreatic Surgery, Kobe University Graduate School of Medicine, Hyogo, Japan; 4 Department of Surgery, Kyoto University Graduate School of Medicine, Kyoto, Japan; 5 Department of Surgery, Fujisawa Shounandai Hospital, Kanagawa, Japan; 6 Department of Public Health, Gunma University Graduate School of Medicine, Gunma, Japan; 7 Department of Surgery and Science, Graduate School of Medical Sciences, Kyushu University, Fukuoka, Japan; 8 Department of Thoracic and Breast Surgery, Faculty of Medicine, Oita University, Oita, Japan; 9 Department of Emergency and Disaster Medicine, Tokyo Medical and Dental University, Tokyo, Japan; 10 Department of Surgery, National Center for Global Health and Medicine, Tokyo, Japan; 11 Department of Surgery, Nagasaki University Graduate School of Biomedical Sciences, Nagasaki, Japan; 12 Department of Upper Gastrointestinal Surgery, Kitasato University School of Medicine, Kanagawa, Japan; 13 Department of Hepato-Biliary-Pancreatic Surgery, Osaka Metropolitan University Graduate School of Medicine, Osaka, Japan; 14 Department of Surgery, Division of Gastroenterological, General and Transplant Surgery, Jichi Medical University, Tochigi, Japan; 15 Department of Gastroenterological Surgery II, Hokkaido University, Sapporo, Japan; 16 Japan Surgical Society, Tokyo, Japan; 17 Department of Gastroenterological Surgery I, Hokkaido University Graduate School of Medicine, Hokkaido, Japan; 18 Department of Breast Oncology, Juntendo University Graduate School of Medicine, Tokyo, Japan; 19 Japan Surgical Society Education Committee, Japan Surgical Society, Tokyo, Japan; 20 Department of Surgery, Tokyo Medical University, Tokyo, Japan

**Correction: Surgery Today** 10.1007/s00595-025-03081-3

In the original publication, two authors’ names were missed. The missing authors’ names are now added in the article. The author group should read as follows:

Daisuke Koike · Kosei Takagi · Keisuke Arai · Yoshiyuki Kiyasu · Takashi Kohmura · Chiaki Suda · Shinkichi Takamori · Wataru Takayama · Mai Nakamura · Masayuki Fukumoto · Yoshiko Yamaoka-Fujikawa · Genki Watanabe · Jun Watanabe · Saseem Poudel · Norihiko Ikeda · Akinobu Taketomi · Mitsue Saito.

The original article has been corrected.

